# Development and internal validation of a model predicting patient-reported shoulder function after arthroscopic rotator cuff repair in a Swiss setting

**DOI:** 10.1186/s41512-023-00156-y

**Published:** 2023-11-07

**Authors:** Thomas Stojanov, Soheila Aghlmandi, Andreas Marc Müller, Markus Scheibel, Matthias Flury, Laurent Audigé

**Affiliations:** 1 Department of Orthopaedic Surgery and Traumatology, University Hospital of Basel, Basel, Switzerland; 2 Research and Development, Shoulder and Elbow Surgery, Schulthess Clinic, Zurich, Switzerland; 3 Department of Clinical Research, Division of Clinical Epidemiology, University Hospital of Basel and University of Basel, Basel, Switzerland; 4 Pediatric Research Center, University Children’s Hospital Basel, Basel, Switzerland; 5 Center for Orthopaedics and Neurosurgery, In-Motion, Wallisellen, Switzerland

**Keywords:** Risk factors, Prognostic factors, Arthroscopy, Rotator cuff tear

## Abstract

**Background:**

Prediction models for outcomes after orthopedic surgery provide patients with evidence-based postoperative outcome expectations. Our objectives were (1) to identify prognostic factors associated with the postoperative shoulder function outcome (the Oxford Shoulder Score (OSS)) and (2) to develop and validate a prediction model for postoperative OSS.

**Methods:**

Patients undergoing arthroscopic rotator cuff repair (ARCR) were prospectively documented at a Swiss orthopedic tertiary care center. The first primary ARCR in adult patients with a partial or complete rotator cuff tear were included between October 2013 and June 2021. Thirty-two potential prognostic factors were used for prediction model development. Two sets of factors identified using the knowledge from three experienced surgeons (Set 1) and Bayesian projection predictive variable selection (Set 2) were compared in terms of model performance using R squared and root-mean-squared error (RMSE) across 45 multiple imputed data sets using chained equations and complete case data.

**Results:**

Multiple imputation using data from 1510 patients was performed. Set 2 retained the following factors: American Society of Anesthesiologists (ASA) classification, baseline level of depression and anxiety, baseline OSS, operation duration, tear severity, and biceps status and treatment. Apparent model performance was R-squared = 0.174 and *RMSE* = 7.514, dropping to R-squared = 0.156, and *RMSE* = 7.603 after correction for optimism.

**Conclusion:**

A prediction model for patients undergoing ARCR was developed using solely baseline and operative data in order to provide patients and surgeons with individualized expectations for postoperative shoulder function outcomes. Yet, model performance should be improved before being used in clinical routine.

**Supplementary Information:**

The online version contains supplementary material available at 10.1186/s41512-023-00156-y.

## Background

Rotator cuff tear is one of the most frequent orthopedic disorders [1]. For patients undergoing arthroscopic rotator cuff repair (ARCR), improvement of shoulder function is one of the main reasons to undergo surgery alongside reduction of shoulder pain or return to sports activities [2].

Outcomes prediction after medical interventions has become a topic of growing interest with the use of prediction models [3]. Indeed, such prediction models can provide patients and surgeons with individualized and evidence-based predictions of surgery success (e.g., by predicting the postoperative shoulder function status or the probability of occurrence of adverse events), supporting healthcare in the decision-making process. Patients may have strong expectations, which should best be tailored to their own health profile and injury characteristics [4]. Such prediction models require prospective and representative outcome data of high-quality and parsimonious development [5].

Despite the rising interest, it is still unclear which prognostic factors are associated with perceived shoulder function outcomes after ARCR [6–12]. The lack of both prospectively collected data and proper methodology to develop and report multivariable prediction models have dramatically impaired the strength of the underlying evidence [13]. The implementation of a local register at a tertiary care clinic in 2013 was the first step towards better documentation and understanding of health outcome data after ARCR [14]. This register has laid the foundation for carrying out a large national ARCR study initiated in 2020 [15]. Both initiatives are set to predict key health outcome data after ARCR such as that recently established for the occurrence of highly prevalent adverse events, such as postoperative shoulder stiffness [16].

### Objectives

Our objectives are (1) to identify potential prognostic factors associated with the postoperative Oxford Shoulder Score (OSS) and (2) to develop and validate a prediction model for postoperative OSS.

## Methods

The Transparent Reporting of a multivariable prediction model for Individual Prognosis Or Diagnosis (TRIPOD) statement was used as a reporting guideline [17].

### Source of data

From January 2010, patients undergoing ARCR in a Swiss tertiary orthopedic clinic were prospectively documented in a register [14]. At the clinic, patient follow-up at 6-month post-surgery comprised various objective and subjective patient-reported outcomes including the Constant-Murley score, Oxford Shoulder Score (OSS), and subjective shoulder value (SSV). The scales were filled out on paper form or via e-mail the week after the clinical examination. An additional 2- to 4-year postoperative evaluation of patient-reported outcomes and level of satisfaction was made by postal questionnaire. All parameters were collected in clinical report forms after the baseline clinical examination or immediately after surgery. Quality checks and data management were done using a Research Electronic Data Capture (REDCap) database [18].

### Participants

Adult patients were included if (1) they had a partial or complete rotator cuff tear that was assessed by magnetic resonance imaging (MRI) and confirmed intraoperatively and (2) underwent ARCR between October 2013 and June 2021. Revision surgeries were excluded as well as contralateral ARCRs in patients with bilateral injuries.

### Treatment and rehabilitation

Shoulder arthroscopy was performed according to internationally standardized procedures with patients in a beach-chair position under general anesthesia [19]. All patients followed a standard 3-phase postoperative physical therapy protocol involving the following: (1) 6 weeks of passive mobilization with an abduction brace (DonJoy UltraSling ER; ORMED GmbH, Freiburg, Germany), (2) 4 to 6 weeks of active-assisted mobilization and coordination training, and (3) specific progressive resistance exercises for the operated shoulder.

### Outcome

For prediction modeling of 6-month outcome, we focused on the OSS, which is a 12-item patient-reported outcome assessing daily functional activities in relation to use of the shoulder [20]. The OSS is a condition-specific questionnaire developed for patients with a degenerative or inflammatory shoulder condition including rotator cuff injuries. The twelve items or questions are answered by the patient independently and address the degree of pain and possible handicaps experienced during activities of daily living within the last 4 postoperative weeks. There are five response categories for each question corresponding to a score ranging from 0 to 4. All scores are then combined to produce a final score ranging from 0 (worst outcome) to 48 (best outcome).

### Prognostic factors

A list of 37 prognostic factors was generated by the primary and senior authors (T. S., L. A.) based on previous systematic reviews [6–12]. Three experienced orthopedic shoulder surgeons (A. M., M. F., M. S.) were asked to independently assess the importance of each prognostic factor using a scale ranging from 0 (not important) to 5 (very important) for the prediction of the 6-month OSS. All 37 factors were documented and available in the local register. We excluded five potential prognostic factors describing redundant information (baseline pain-related question from the Constant-Murley score, general health status, rotator cuff tear pattern, the extent of the rotator cuff tear, and the baseline pain-related question from a visual analog scale).

Thirty-two potential prognostic factors were finally retained for development of the prediction model including the following: 18 patients and disease-related parameters collected at baseline (age at surgery, sex, body mass index, American Society of Anesthesiologists (ASA) physical status classification, dominance of affected side, smoking status at surgery, three preoperative treatment variables (medication, physiotherapy, and steroid infiltrations), traumatic onset, symptom duration, level of depression and anxiety using the European Quality-of-Life 5 Dimensions 5 Level (EQ-5D-5L) scale [21] and baseline functional scales (OSS, Constant-Murley score, range of parameters (flexion, abduction, external rotation)), and muscle strength) and 14 operative findings and details collected during the surgery (supraspinatus tear, subscapularis tear, infraspinatus tear, tear severity, level of fatty infiltration, tendon degeneration, tendon delamination, operation duration, number of anchors used, number of threads used, acromioclavicular joint resection, acromioplasty, capsulotomy, and biceps tendon status and treatment).

### Post hoc sample size calculation

An R package developed by Riley et al. [22] was used to estimate the necessary sample size for the development of a multivariable prediction model for continuous outcomes (*pmsampsize* package). The estimation required an expected R-squared value for the future prediction model of 0.2, a shrinkage factor of 0.9, a multiplicative margin of error of 1.1, the number of parameters to be assessed during the multivariable prediction model development of 32, and an average outcome value in the population of interest (i.e., *intercept*) of *40* with its standard deviation (*SD*) of 8. The necessary sample size estimated at 1199 assumed a 0.05 acceptable difference in apparent and adjusted R-squared.

### Missing data

Considering our data set showed variable missing data rates and that these missing values were missing at random, multiple imputation of missing data was performed using chained equations [23] with 45 datasets based on all the available information (including the date of surgery).

### Statistical analyses

All analyses were performed using R [24].

#### Type of model used

Linear regression models were fitted using ordinary least square estimation. Univariable regression coefficients and their 95% confidence intervals (CI) were reported and compared between multiple imputed datasets and complete case data.

#### Prognostic factor handling

Following the ten principles to strengthen prognosis research [25], continuous prognostic factors were kept continuous as far as possible. Based on univariable regression models, second-order polynomial transformations of continuous predictors were tested to account for the nonlinear association with postoperative OSS. For categorical variables, an attempt was made to avoid sparse categories.

#### Model building procedures

Two sets of factors were compared: “Set 1” regrouped the eleven factors estimated by surgeons as having the best predictive ability ( including the baseline OSS value), and “Set 2” was composed of the variables identified using Bayesian projection predictive variable selection using the *projpred* package [26]. Five-fold cross validation was performed for variable selection based on the complete case data. The set of variables with the best predictive ability (in terms of root-mean-square error (RMSE)) was then identified.

#### Model performance

The predictive performance of the two sets of factors were compared in terms of R-squared and RMSE. Internal bootstrap validation was performed to estimate optimism-corrected model performance using 500 repetitions [27]. Regression coefficients were presented in the main text for the model with the best optimism-corrected predictive ability.

### Patient and public involvement in research statement

No patient or member of the public was involved in the design of this study. Patients directly filled out the Oxford Shoulder Score based on their own experience after the surgery.

## Results

### Participants

Overall, 1555 patients were assessed for eligibility between October 2013 and June 2021. After application of our eligibility criteria, 45 patients were excluded (1 patient owed to the group IV of ASA classification; 44 were scheduled for a revision surgery) leading to a set of 1510 patients. Complete-case data were available for 712 patients. The percentage of missing values for specific variables ranged from 0 to 45.4% (Table 1).

**Table 1:** Study population characteristics

Characteristics	Prognostic value^1^	Missing, %	Multiple imputed data^2^		Complete-case data^2^	
			**Distribution**^**3**^	**Univariable linear regression model**^**4**^	**Distribution**	**Univariable linear regression model**
**Patient and disease-related factors (*N* = 18)**						
Age at surgery, in years	6.5	0	59 (53, 65)	0.08 [0.03; 0.12]	59 (52, 64)	0.05 [− 0.01; 0.12]
Male sex	5	0	613 (41%)	− 1.93 [− 2.78; − 1.09]	300 (42%)	− 1.63 [− 2.87; − 0.39]
Body mass index (BMI), in kg/m^2^	6.5	0.7	26 (23, 28)	− 0.19 [− 0.29; − 0.1]	26 (23, 29)	− 0.23 [− 0.37; − 0.08]
ASA classification	5	0.1				
I			383 (25%)	Ref	176 (25%)	Ref
II			941 (62%)	− 0.79 [− 1.76; 0.19]	464 (65%)	− 0.35 [− 1.78; 1.09]
III			186 (12%)	− 2.91 [− 4.35; − 1.46]	72 (10%)	− 4.18 [− 6.45; − 1.9]
Dominant side operated	3	0.9	1019 (67%)	− 0.36 [− 1.26; 0.54]	495 (70%)	− 0.96 [− 2.3; 0.37]
Smoker at surgery	12.5	0.3	242 (16%)	− 1.7 [− 2.84; − 0.56]	107 (15%)	− 2.02 [− 3.73; − 0.31]
Preoperative medication	9	45.4	1105 (73%)	− 0.48 [− 1.69; 0.73]	563 (79%)	− 0.46 [− 1.97; 1.05]
Preoperative physiotherapy	6	45.4	684 (45%)	− 0.59 [− 1.64; 0.46]	319 (45%)	− 0.6 [− 1.84; 0.63]
Preoperative steroid infiltrations	7.5	1.9				
None			1193 (79%)	Ref	445 (63%)	Ref
One			165 (11%)	− 1.28 [− 2.63; 0.08]	141 (20%)	− 0.89 [− 2.47; 0.68]
Two or more			152 (10%)	− 3.28 [− 4.71; − 1.84]	126 (18%)	− 2.39 [− 4.04; − 0.74]
Traumatic onset	11	0	784 (52%)	0.79 [− 0.05; 1.62]	374 (53%)	0.48 [− 0.75; 1.71]
Symptom duration	8	1.2				
Less than a month			157 (10%)	Ref	69 (9.7%)	Ref
One to 3 months			286 (19%)	− 0.39 [− 2.01; 1.22]	122 (17%)	− 0.52 [− 2.98; 1.95]
Three to 6 months			344 (23%)	− 0.5 [− 2.06; 1.07]	157 (22%)	− 0.67 [− 3.03; 1.69]
Six months to 1 year			298 (20%)	− 1.51 [− 3.11; 0.09]	153 (21%)	− 2.25 [− 4.62; 0.12]
More than a year			425 (28%)	− 1.69 [− 3.21; − 0.17]	211 (30%)	− 1.51 [− 3.78; 0.76]
Level of depression and anxiety (EQ-5D-5L)	12.5	1.3				
Not anxious/depressed			995 (66%)	Ref	461 (65%)	Ref
A bit anxious/depressed			329 (22%)	− 1.97 [− 2.98; − 0.97]	166 (23%)	− 2.26 [− 3.71; − 0.81]
At least moderately anxious/depressed			186 (12%)	− 6.38 [− 7.64; − 5.12]	85 (12%)	− 5.21 [− 7.1; − 3.31]
Baseline constant score	8	29.7	58 (44, 68)	0.11 [0.08; 0.14]	57 (45, 67)	0.1 [0.06; 0.13]
Baseline flexion, in 10-degree unit	12	24.2	15 (14, 17)	0.29 [0.1; 0.47]	15 (14, 16)	0.18 [− 0.06; 0.42]
Baseline abduction, in 10-degree unit	11	24.4	15 (12, 16)	0.17 [0.01; 0.32]	15 (12, 16)	0.11 [− 0.09; 0.32]
Baseline external rotation, in 10-degree unit	6	24.6	6 (4, 7)	0.01 [-0.29; 0.32]	5 (4, 6)	− 0.05 [− 0.43; 0.33]
Baseline muscle strength in abduction, in kg	10	25.1	4 (1, 7)	0.23 [0.1; 0.36]	4 (2, 7)	0.21 [0.03; 0.38]
Baseline Oxford Shoulder Score	15	0	29 (23, 35)	0.33 [0.28; 0.38]	29 (22, 34)	0.31 [0.24; 0.38]
**Operative findings and details (*N* = 14)**						
Supraspinatus tear	6.5	0	1428 (95%)	0.62 [− 1.22; 2.46]	673 (95%)	1.4 [− 1.3; 4.1]
Subscapularis tear	12	0	485 (32%)	− 0.13 [− 1.03; 0.76]	218 (31%)	0.26 [− 1.07; 1.6]
Infraspinatus tear	8	0	404 (27%)	0.89 [− 0.05; 1.83]	174 (24%)	0.65 [− 0.78; 2.08]
Tear severity (Gerber classification)	11	0				
Partial tear			332 (22%)	Ref	155 (22%)	Ref
Single full tear			572 (38%)	0.49 [− 0.63; 1.61]	283 (40%)	0.16 [− 1.47; 1.8]
Two or three tendons (only one full)			314 (21%)	0.26 [− 1.02; 1.53]	141 (20%)	− 0.46 [− 2.37; 1.45]
Massive tear			292 (19%)	0.77 [− 0.53; 2.07]	133 (19%)	1.38 [− 0.56; 3.32]
Level of fatty infiltration	10	26.7				
Level 0			761 (50%)	Ref	379 (53%)	Ref
Level 1			628 (42%)	− 1.56 [− 2.51; − 0.61]	281 (39%)	− 1.12 [− 2.4; 0.17]
Level 2			121 (8.0%)	− 1.57 [− 3.33; 0.19]	52 (7.3%)	− 1.34 [− 3.76; 1.09]
Tendon degeneration	7	0	839 (56%)	0.16 [− 0.68; 1]	372 (52%)	0.27 [− 0.96; 1.5]
Tendon delamination	7.5	0	520 (34%)	− 1.22 [− 2.1; − 0.35]	258 (36%)	− 0.85 [− 2.13; 0.42]
Operation duration, in minutes	8	0.1	75 (60, 94)	− 0.02 [− 0.04; − 0.01]	73 (59, 94)	− 0.03 [− 0.06; − 0.01]
Number of anchors used	7	0				
0–2			238 (16%)	Ref	114 (16%)	Ref
3–4			1069 (71%)	− 0.06 [− 1.23; 1.1]	488 (69%)	− 1.01 [− 2.71; 0.7]
5 +			203 (13%)	− 0.23 [− 1.78; 1.32]	110 (15%)	− 0.24 [− 2.43; 1.95]
Number of threads	7	0	4 (2, 5)	0.14 [− 0.09; 0.37]	3 (2, 4)	0.26 [− 0.09; 0.61]
Acromioclavicular joint resection	2	0	323 (21%)	− 1.76 [− 2.78; − 0.75]	139 (20%)	− 1.7 [− 3.25; − 0.16]
Acromioplasty	4	0	1365 (90%)	1.03 [− 0.38; 2.45]	643 (90%)	− 0.19 [− 2.26; 1.89]
Capsulotomy	8	0	116 (7.7%)	− 0.45 [− 2.02; 1.12]	66 (9.3%)	− 0.06 [− 2.18; 2.05]
Biceps status & treatment	6	0				
No treatment			236 (16%)	Ref	110 (15%)	Ref
Tenotomy			287 (19%)	− 2.02 [− 3.44; − 0.6]	175 (25%)	− 1.52 [− 3.5; 0.47]
Tenodesis			877 (58%)	0.21 [− 0.97; 1.39]	391 (55%)	0.48 [− 1.29; 2.24]
Already ruptured/treated			110 (7.3%)	− 0.24 [− 2.11; 1.62]	36 (5.1%)	0.06 [− 3.08; 3.2]
6-month Oxford Shoulder Score			43 (37, 46)		42 (37, 45)	

^1^Prognostic values were summed based on the ratings of three experienced orthopedic surgeons. ^2^Median (interquartile range); n (%). ^3^Average distribution across the multiple imputed datasets (*N* = 45). ^4^Pooled regression coefficients across the multiple imputed datasets (*N* = 45)

Comparisons between thirty-two prognostic factor distributions across average multiple imputed data and available complete-case data are summarized in Table 1. Univariable associations between each candidate prognostic factor and the 6-month OSS are also reported. No prognostic factors showed nonlinear univariable associations with the postoperative outcome, even after multiple imputation.

### Model development

#### Outcome data

After multiple imputation, the median baseline OSS was 29 (interquartile range (IQR) 23, 35) for the whole population and increased to 43 points (*IQR* 37, 48) 6 months after the surgery (Table 1). The 6-month OSS values were recorded between 4 and 11 months after the surgery. Overall, 181 patients achieved the maximum OSS value at 6 months (12%). For 167 patients (11%), the change between baseline and the 6-month postoperative OSS was below or equal to 0.

### Model specification and comparison

Set 1 and Set 2 were composed of 11 and 7 prognostic factors, respectively (see Additional file 1 and Table 3). After correction for optimism, Set 2 had better model performance than any other model after multiple imputation with R-squared (0.156) and RMSE (7.603) (Table 2).

**Table 2:** Multivariable model comparison

		**Apparent**		**Optimism corrected**	
**Model**	**Dataset**	**R-squared**	**RMSE**	**R-squared**	**RMSE**
Set 1	Complete case	0.131	7.774	0.092	7.961
Set 1	Multiple imputation	0.162	7.565	0.146	7.63
Set 2	Complete case	0.162	7.634	0.123	7.818
**Set 2**	**Multiple imputation**	**0.174**	**7.514**	**0.156**	**7.603**

### Model presentation

For Set 2, adjusted regression coefficients were similar across multiple imputed and complete-case data (Table 3). This model included data collected during baseline examinations and operative details.

**Table 3:** Linear multivariable model for Set 2

Characteristics	Multiple imputed data^1^	Complete-case data
Intercept	35.56 [33.15; 37.97]	36.19 [32.6; 39.77]
**Patient and disease-related factors (*N* = 4)**		
ASA classification		
I	Ref	Ref
II	− 0.11 [− 1.03; 0.8]	0.31 [− 1.08; 1.69]
III	− 1.59 [− 2.95; − 0.22]	− 2.57 [− 4.79; − 0.35]
Symptom duration		
Less than a month	Ref	Ref
One to 3 months	− 1.02 [− 2.51; 0.47]	− 1.42 [− 3.74; 0.9]
Three to 6 months	− 1.65 [− 3.12; − 0.18]	− 1.68 [− 3.92; 0.56]
Six months to 1 year	− 3.07 [− 4.58; − 1.56]	− 3.36 [− 5.62; − 1.1]
More than a year	− 2.68 [− 4.12; − 1.24]	− 2.31 [− 4.47; − 0.14]
Level of depression and anxiety (EQ-5D-5L)		
Not anxious/depressed	Ref	Ref
A bit anxious/depressed	− 1.11 [− 2.07; − 0.15]	− 1.3 [− 2.7; 0.11]
At least moderately anxious/depressed	− 4.08 [− 5.31; − 2.84]	− 2.91 [− 4.78; − 1.05]
Baseline Oxford Shoulder Score	0.3 [0.25; 0.35]	0.28 [0.2; 0.35]
**Operative findings and details (*N* = 3)**		
Operation duration, in minutes	− 0.03 [− 0.04; − 0.01]	− 0.04 [− 0.07; − 0.02]
Tear severity (Gerber classification)		
Partial tear	Ref	Ref
Single full tear	0.84 [− 0.22; 1.89]	0.84 [− 0.73; 2.41]
Two or three tendons (only one full)	0.93 [− 0.32; 2.18]	0.66 [− 1.25; 2.56]
Massive tear	1.83 [0.48; 3.18]	2.9 [0.88; 4.92]
Biceps status & treatment		
No treatment	Ref	Ref
Tenotomy	− 0.78 [− 2.18; 0.62]	− 0.31 [− 2.33; 1.71]
Tenodesis	0.48 [− 0.71; 1.67]	1.24 [− 0.55; 3.04]
Already ruptured/treated	0.07 [− 1.71; 1.86]	0.99 [− 2.08; 4.05]

^1^Pooled regression coefficients across the multiple imputed datasets (*N* = 45)

## Discussion

We developed a prediction model for shoulder function 6 months after an ARCR. Lack of important prognostic factors might explain the improvable model performance. We used robust techniques for the variables selection. Seven patients, disease, and operative factors were retained in the model showing the best model performance after multiple imputation. Our model development highlights the importance to take into account baseline and operative details when the aim is to predict postoperative course of functional status after an orthopedic intervention. Yet, the model performance suggests a model update before implementation in clinical practice, which is planned with the implementation of the ARCR_Pred data [15].

With sound model performance, such models might be useful for clinicians and policymakers with a primary focus on patient-centered healthcare. Alongside individual expectations of shoulder function outcomes held by the surgeon, such models provide useful insight into the associations between prognostic factors and patient-reported outcomes. This modeled evidence might then be discussed with patients during clinical examinations to forecast their possibilities of improvements after surgery based on informed consideration of a set of pre-established factors. The limitations encountered in this study can be addressed by stronger national support in the development of clinical registers, which form the basis for value-based health care in orthopedic surgery [15, 16].

In prognostic research studies, associations between a given factor and an outcome have to be replicated across different studies in order to classify the factor as one with potential prognostic importance [28]. In our recent systematic review, we identified that low preoperative functional status was associated with greater improvement in PROMs, which seems logical given that patients with poorer outcome scores are expected to have a greater change in outcome [29]. In this case, the type of modeled outcome has substantial importance on the direction in which the associations occur. In the present work, patients with a higher preoperative OSS had better a 6-month score. Optimization of preoperative functional scores might therefore be important in order to expect better postoperative outcomes. This could be achieved by implementing a better selection of the patients undergoing an ARCR (i.e., patients below a certain score could first be optimized with preoperative non-interventional treatment).

Frangiamore et al. recently developed models for various postoperative outcomes also impacted by substantial ceiling and floor effects, which they addressed with the use of proportional odds ordinal logistic regression [30]. Their set comprised twelve factors based on clinical rationale (age at surgery, sex, worker’s compensation status, previous cuff repair, tear size, critical shoulder angle, baseline outcome values, length of follow-up, fatty infiltration, tear shape, tendon stump length, and multiple tendon involvement). Unfortunately, this working group did not report a model performance indicator, which leaves us with a lack of information for comparison. In a recent review, we identified the need for researchers to use adequate reporting guidelines when reporting multivariable prediction models [31].

### Strengths

This study followed the most recent reporting guidelines along with the availability of the code, which makes this study reproducible and transparent. We included various patient, disease, and procedure-related factors and asked experienced surgeons to assess their predictive ability. We also implemented robust variable selection techniques, such as the Bayesian projection predictive variable selection.

### Limitations

The lack of other important predictors (including postoperative management or peri-operative factors) in our model development may be partially responsible for our inability to accurately make forecasts for patients with extreme postoperative values. For instance, the occurrence of adverse events such as repair failure, neurological injury, persistent pain, or shoulder stiffness might dramatically affect our predictions. It might therefore be important to accurately identify and monitor patients with postoperative adverse events. This study also suffered from the absence of proper external validation.

## Conclusions

Before being implemented in clinical practice, our prediction model should show better model performance. We believe the identification of a new set of variables using the ARCR_Pred data might help in that regard by using a variety of new parameters encompassing those of preoperative and/or peri-operative management [15].

### Supplementary Information

**Additional file 1.** Linear multivariable model for Set 1.

## Data Availability

The datasets and code used during the current study are available from the corresponding author on reasonable request.

## Abbreviations

AIC: Akaike’s information criterion
ARCR: Arthroscopic rotator cuff repair
OSS: Oxford Shoulder Score
RMSE: Root-mean-squared error
SD: Standard deviation
TRIPOD: Transparent reporting
