# Supplementary material for: Development and internal validation of a model predicting patient-reported shoulder function after arthroscopic rotator cuff repair in a Swiss setting
Source: Diagn Progn Res. 2023 Nov 7;7:21. doi: 10.1186/s41512-023-00156-y (PMC10629040; doi:10.1186/s41512-023-00156-y)
Supplement: Supplementary file 1 — Additional file 1. Linear multivariable model for Set 1. [file 41512_2023_156_MOESM1_ESM.docx]

| **Characteristics** | **Multiple imputed data^1^** | **Complete-case data** |
| --- | --- | --- |
| (Intercept) | 31.6 [28.33; 34.87] | 32.53 [28.03; 37.03] |
| **Patient and disease-related factors (N = 8)** |  |  |
| Smoker at surgery | -1.26 [-2.32; -0.2] | -1.61 [-3.24; 0.02] |
| Preoperative medication | 0.45 [-0.76; 1.66] | 0.47 [-1; 1.94] |
| Traumatic onset | 0.61 [-0.19; 1.41] | 0.18 [-1.02; 1.37] |
| Level of depression and anxiety (EQ5D5L) |  |  |
| Not anxious/depressed | Ref. | Ref. |
| A bit anxious/depressed | -1.25 [-2.21; -0.28] | -1.57 [-2.98; -0.16] |
| At least moderately anxious/depressed | -4.25 [-5.5; -3] | -3.35 [-5.24; -1.45] |
| Baseline flexion, in 10-degrees unit | 0.04 [-0.21; 0.29] | -0.03 [-0.39; 0.34] |
| Baseline abduction, in 10-degree unit | -0.02 [-0.24; 0.2] | -0.03 [-0.34; 0.28] |
| Baseline muscle strength in abduction, in kg | -0.03 [-0.18; 0.13] | -0.03 [-0.23; 0.18] |
| Baseline Oxford Shoulder Score | 0.29 [0.24; 0.35] | 0.29 [0.21; 0.37] |
|  |  |  |
| **Operative findings and details (N = 3)** |  |  |
| Subscapularis tear | -0.36 [-1.32; 0.59] | 0.19 [-1.29; 1.66] |
| Level of fatty infiltration |  |  |
| Level 0 | Ref. | Ref. |
| Level 1 | -1.42 [-2.34; -0.5] | -1.22 [-2.51; 0.07] |
| Level 2 | -2.09 [-3.89; -0.29] | -2.07 [-4.51; 0.37] |
| Tear severity (Gerber classification) |  |  |
| Partial tear | Ref. | Ref. |
| Single full tear | 0.98 [-0.09; 2.06] | 0.92 [-0.68; 2.52] |
| Two or three tendons (only one full) | 1.12 [-0.16; 2.41] | 0.5 [-1.48; 2.47] |
| Massive tear | 2.17 [0.78; 3.57] | 2.52 [0.44; 4.6] |
|  |  |  |
| 1: Pooled regression coefficients across the multiple imputed datasets (N = 45) | | |

**Additional File 1.** Linear multivariable model for Set 1
